# Spatial training preserves associative memory capacity with augmentation of dendrite ramification and spine generation in Tg2576 mice

**DOI:** 10.1038/srep09488

**Published:** 2015-03-30

**Authors:** Xia Jiang, Gao-Shang Chai, Zhi-Hao Wang, Yu Hu, Xiao-Guang Li, Zhi-Wei Ma, Qun Wang, Jian-Zhi Wang, Gong-Ping Liu

**Affiliations:** 1 Department of Pathophysiology, Key Laboratory of Ministry of Education for Neurological Disorders, the School of Basal Medicine, Tongji Medical College, Huazhong University of Science and Technology, Wuhan, 430030. P. R. China; 2 Department of Pathology, Hubei University of Chinese Medicine, Wuhan, 430065. P. R. China; 3 Department of Basic Medicine, Wuxi Medical School, Jiangnan University, Wuxi, Jiangsu Province, 214122, P. R. China; 4 Co-innovation Center of Neuroregeneration, Nantong University, Nantong, JS 226001, China

## Abstract

Alzheimer's disease (AD) is the most common neurodegenerative disorder and there is currently no efficient cure for this devastating disease. Cognitive stimulation can delay memory loss during aging and in patients with mild cognitive impairment. In 3 × Tg-AD mice, training decreased the neuropathologies with transient amelioration of memory decline. However, the neurobiological mechanisms underlying the learning-improved memory capacity are poorly understood. Here, we found in Tg2576 mice spatial training in Morris water maze (MWM) remarkably improved the subsequent associative memory acquisition detected by contextual fear conditioning. We also found that spatial training enhanced long term potentiation, dendrite ramification and spine generation in hippocampal dentate gyrus (DG) and CA1 neurons at 24 h after the training. In the molecular level, the MWM training remarkably activated calcium/calmodulin-dependent protein kinase II (CaMKII) with elevation of glutamate AMPA receptor GluA1 subunit (GluA1), postsynaptic density protein 93 (PSD93) and postsynaptic density protein 95 (PSD95) in the hippocampus. Finally, the training also significantly ameliorated AD-like tau and amyloid pathologies. We conclude that spatial training in MWM preserves associative memory capacity in Tg2576 mice, and the mechanisms involve augmentation of dendrite ramification and spine generation in hippocampus.

Alzheimer's disease (AD) is the most common neurodegenerative disorder characterized clinically by spatial memory loss in early stage of the onset. Accumulation of β-amyloid and hyperphosphorylated microtubule-associated protein tau are the major pathological hallmarks in the AD brains. The mechanisms underlying neurodegeneration are not fully elucidated, and there is currently no proven, effective disease-modifying therapy for this devastating disorder.

Low education increases risk for AD, and cognitive stimulating therapy improves the memory functions with the same efficacy as galantamine or tacrine^1^. Studies also show that participation in cognitively stimulating activities, such as reading, is associated with a reduced risk of dementia^2^,^3^,^4^ and a reduced risk of amnestic mild cognitive impairment (aMCI)^5^,^6^. Different types of cognitive training programs can also improve the cognitive function in elder population^6^,^7^. These studies strongly suggest that cognitive stimulation can preserve memory capacities in AD and aMCI patients. However, the neurobiological mechanisms underlying the training-induced memory improvement are largely unknown.

Morris water maze training was reported to reduce the amyloid load and tau hyperphosphorylation with improvement of memory in 3 × Tg-AD mice^8^. Although the amyloid plaques and tau hyperphosphorylation/neurofibrillary tangle formation are the primary characteristics of pathology in AD, these pathological changes are not strongly correlated with the cognitive decline of the patients^9^,^10^. On the other hand, the synaptic strength, which is influenced by dendrite ramification and spine generation/plasticity, play a crucial role in learning and memory^11^,^12^. Loss of synapse and spine in various brain areas strongly correlates with the clinical scores of dementia of AD^13^,^14^,^15^. The dendrite ramification and spine generation are regulated by the expression of postsynaptic proteins, such as postsynaptic density protein 93 (PSD93), postsynaptic density protein 95 (PSD95), and calcium/calmodulin-dependent protein kinase II (CaMKII)^16^,^17^,^18^,^19^.

In the present study, we trained Tg2576 mice, a widely used AD-like model that show memory deficits at 8 m old. The mice were received 6 consecutive days training in Morris water maze (MWM) given at ~8 m, then the effects of spatial training on the memory capacity were measured using contextual fear conditioning. We found that spatial training significantly improved the subsequent memory acquisition and maintenance in the mice. This cognitive improvement was associated with the enhanced long term potentiation (LTP) and the CaMKII-associated remodeling of dendritic plasticity.

## Results

### Spatial training preserves the recent and remote memory capacities in Tg2576 mice

**Figure 1:**
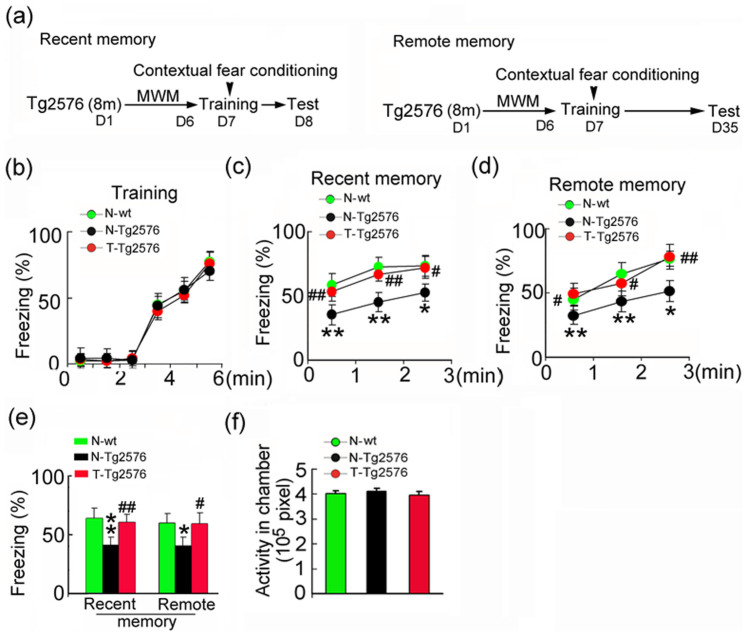
Spatial training ameliorates hippocampus-dependent memory deficits in Tg2576 mice. (a) Schematics show experimental procedure: Tg2576 mice received 6 days Morris water maze (MWM) training at ~8 m. Then, the recent and remote memory was retrieved at 24 h or 28 days by contextual fear conditioning. (b–e) Contextual fear conditioning: The freezing response during the fear training (B), the retrieval test of freezing response measured at 24 h after fear training (recent memory) (c), the retrieval test of freezing response measured at 28 days after fear training (remote memory) (d), quantitative analyses of average freezing response over the 3 min session (e). (f) The activity during the initial 3 min exposure to the conditioning chamber. Not trained wild type littermates (N-wt); not trained Tg2576 control mice (N-Tg2576); the trained Tg2576 mice (T-Tg2576). Data were expressed as mean ± SD (n = 10 ~ 12 each group). One-way ANOVA followed by post hoc tests (LSD) was used. *, *p* < 0.05; **, *p* < 0.01 *vs* N-wt; #, *p* < 0.05; ##, *p* < 0.01 *vs* N-Tg576. (c) N-Tg2576 *vs* N-wt: p_1_ = 0.002, p_2_ = 0.004, p_3_ = 0.023; T-Tg2576 *vs* N-Tg2576: p_1_ = 0.006, p_2_ = 0.007, p_3_ = 0.030; (d) N-Tg2576 *vs* N-wt: p_1_ = 0.033, p_2_ = 0.006, p_3_ = 0.002; T-Tg2576 *vs* N-Tg2576: p_1_ = 0.012, p_2_ = 0.031, p_3_ = 0.001; (e) N-Tg2576 *vs* N-wt: p_recent_ = 0.002, p_remote_ = 0.010; T-Tg2576 *vs* N-Tg2576: p_recent_ = 0.039, p_remote_ = 0.017.

Tg2576 mice were trained for 6 consecutive days (T-Tg2576), while the control Tg2576 (N-Tg2576) and the non-Tg littermates (N-wt) were handled with swimming activity but without training (Fig. 1a). The three groups of mice had experienced same swimming time in the maze, and no significant difference in swimming distances was detected (Fig. S1), suggesting no motor dysfunction. At 24 h after the last training, the recent and remote memory was assessed by contextual fear conditioning for associative memory that involves amygdale and the hippocampus^20^. We found that the hippocampus-dependent MWM training significantly increased the freezing response of Tg2576 mice in retrieval tests at 24 h after spatial training (recent memory) (Fig. 1c and e). These improvements were still significant at 28 day (remote memory) (Fig. 1d and e). No difference was seen between the trained and not-trained groups in the freezing behavior during fear training (Fig. 1b). The animals also did not show any motor impairment measured by pre-foot shock activity (Fig. 1f). These data suggest that spatial training can improve memory acquisition and maintenance in Tg2576 mice.

### Spatial training enhances hippocampal LTP

**Figure 2:**
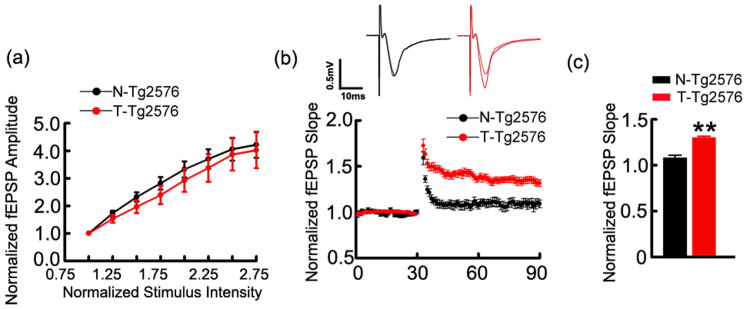
Spatial training potentiates LTP. (a) Input-output curve was similar in the untrained and trained Tg2576 mice. (b, c) The normalized fEPSP slop at the medial perforant path to granule cells synapse in ~9 m old Tg2576 mice. Data were expressed as mean ± SD (n = 3 ~ 4 each group), Student's t test was used. (c) p = 0.0004.

By using electrophysiological recording in dentate gyrus (DG) granule cells in acute hippocampal slices, we found that spatial training did not change the baseline synaptic response evaluated by input/output (I/O) curves of field excitatory postsynaptic potentials (fEPSP) slope in Tg2576 mice (Fig. 2a). However, during the second half of baseline recordings co-terminating with the end of high frequency stimulation, a significantly augmented LTP was detected in the trained groups compared with the not-trained Tg2576 mice (Fig. 2b and c), suggesting an increased synaptic transmission induced by spatial training.

### Spatial training remodels dendritic complexity with increased spine density and plasticity

**Figure 3:**
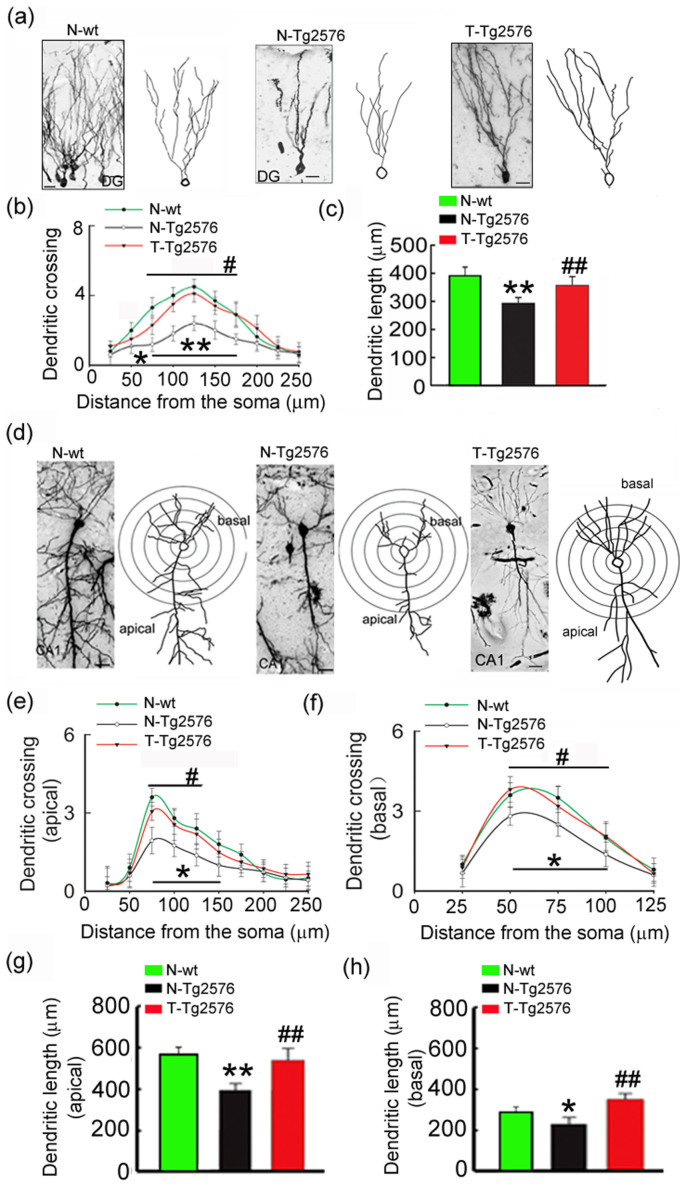
Spatial training remodels dendritic complexity. (a) Dendritic arbors measured by Golgi staining and reconstruction of the dendritic arbors in DG granular neurons of not trained wild type littermates (N-wt, 8 m old); not trained Tg2576 control mice (N-Tg2576, 8 m old); and the trained Tg2576 mice (T-Tg2576, 8 m old). (b, c) Sholl analyses of the numbers of dendritic points and quantification of total dendritic length in DG granular neurons. (d) Dendritic arbors shown by Golgi staining and reconstruction of the dendritic arbors in CA1 pyramidal neurons. (E–H) Sholl analyses of the numbers of dendritic crossing (e, f) and quantification of dendritic length of apical and basal dendritic arbors (g, h) of CA1 pyramidal neurons. Data were expressed as mean ± SD (n = 3 each group). One-way ANOVA followed by post hoc tests (LSD) was used. *, *p* < 0.05; **, *p* < 0.01 *vs* N-wt; #, *p* < 0.05; ##, *p* < 0.01 *vs* N-Tg576. Bar = 20 μm. (b) N-Tg2576 *vs* N-wt: p_50_ = 0.023; p_75_ < 0.001, p_100_ = 0.001, p_125_ = 0.001, p_150_ = 0.004, p_175_ = 0.004; T-Tg2576 *vs* N-Tg2576: p_75_ = 0.032, p_100_ = 0.011, p_125_ = 0.004, p_150_ = 0.014, p_175_ = 0.004. (c) N-Tg2576 *vs* N-wt: p = 0.008; T-Tg2576 *vs* N-Tg2576: p = 0.003. (e) N-Tg2576 *vs* N-wt: p_75_ < 0.001, p_100_ = 0.010, p_125_ = 0.006, p_150_ = 0.009; T-Tg2576 *vs* N-Tg2576: p_75_ = 0.015, p_100_ = 0.042, p_125_ = 0.006. (f) N-Tg2576 *vs* N-wt: p_50_ = 0.002; p_75_ = 0.005, p_100_ = 0.030; T-Tg2576 *vs* N-Tg2576: p_50_ < 0.001, p_75_ = 0.043, p_100_ = 0.013. (g) N-Tg2576 *vs* N-wt: p = 0.001; T-Tg2576 *vs* N-Tg2576: p = 0.001. (H) N-Tg2576 *vs* N-wt: p = 0.027; T-Tg2576 *vs* N-Tg2576: p < 0.001.

To explore the mechanisms underlying the training-induced preservation of memory capacity, we measured the dendritic arbors in hippocampal DG and CA1 regions by using Golgi impregnation and concentric circle analysis^21^. We found that the untrained Tg2576 mice had shorter dendrite length and fewer dendritic crossings in both DG and CA1 compared with the age-matched non-tg littermates (Fig. 3b, c, e–h). Spatial training significantly increased the length and the number of dendritic branches extending beyond 75–175 μm from the cell soma in DG (Fig. 3a–c). In CA1 pyramidal neurons, the increased dendritic branches extending beyond 50–100 μm (basal) or 75–125 μm (apical) from the cell soma were also detected in trained Tg2576 mice (Fig. 3d–h).

**Figure 4:**
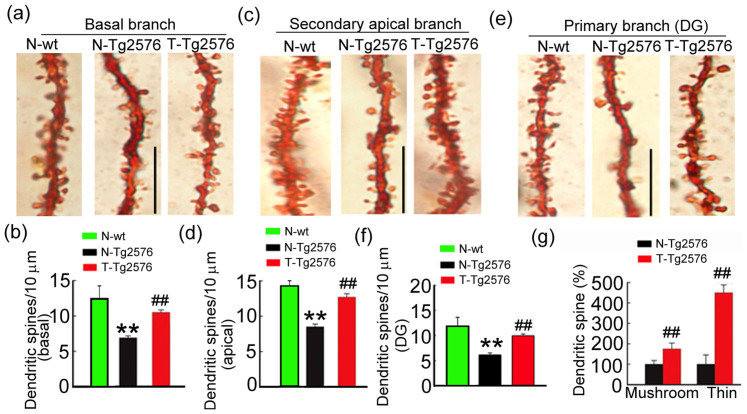
Spatial training increases spine density in hippocampal DG and CA1 neurons. The representative photomicrographs and the quantification of the secondary apical branches and the primary basal branches of hippocampal CA1 pyramidal neurons (a–d), the representative photomicrographs and the quantification of primary branches in hippocampal DG granular neurons (e, f). Bar = 10 μm. (g) The density of the mushroom- or thin-shaped spines was calculated in Tg2576 mice. Data were expressed as mean ± SD (n = 3 ~ 5 each group, at least 36 neurons were analyzed for each animal). One-way ANOVA followed by post hoc tests (LSD) was used. **, *p* < 0.01 *vs* N-wt; ##, *p* < 0.01 *vs* N-Tg576. (b) **, *p* < 0.001; ## *p* = 0.001. (d) **, *p* < 0.001; ##, *p* < 0.001. (f) **, *p* = 0.002; ##, *p* = 0.004. (g) p_mushroom_ = 0.008, p_thin_ = 0.001.

We further refined the analysis by investigating whether spatial training affects the formation of dendritic spines which indirectly reflect the presence of synaptic inputs^22^. By Golgi staining and morphological analysis, we found that the spine density in DG and CA1 neurons was significantly reduced in ~9 m-old Tg2576 mice when compared with the age-matched non-tg littermates (Fig. 4a–f). After spatial training, the spine number in primary and secondary branches of CA1 and DG neurons were significantly increased in Tg2576 mice (Fig. 4a–f). By dividing the spines into two categories based on the head and neck size^23^,^24^, we observed that both the thin and mushroom spines were increased in DG neurons after training. More interestingly, the number of thin spines was respectively 2.5- and 2.3-fold of the mushroom spines in Tg2576 (Fig. 4g), which supports the conclusion of increased post-synaptic plasticity induced by spatial training.

### Spatial training increases the expression of GluA1, PSD93 and PSD95 with a significant upregulation of phosphorylated CaMKII

**Figure 5:**
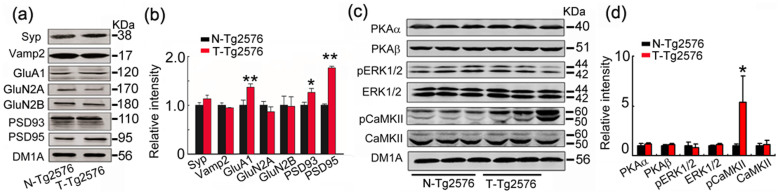
Spatial training increases the levels of specific postsynaptic-associated proteins and phosphorylated CaMKII. Expression of pre- and post-synaptic proteins (a, b), and PKAα, PKAβ, ERK1/2 and phosphorylated ERK1/2 (pERK1/2), CaMKII and phosphorylated CaMKII (pCaMKII) (c, d) in the hippocampal extracts of Tg2576 mice with or without spatial training were measured by Western blotting and quantitative analyses. Blot images were cropped for comparison. DM1A was used as a loading control in each sample. The optical density of bands was quantified by Gel-Pro Analyzer 4 (Toyobo, Osaka, Japan) software. Data were expressed as mean ± SD (n = 4 each group). Student's t test was used. *, *p* < 0.05; **, *p* < 0.01 vs N-Tg576. (b) p_GluA1_ < 0.001, p_PSD93_ = 0.034, p_PSD95_ = 0.002. (d) p_pCaMKII_ = 0.017.

To explore the molecular mechanisms underlying the spatial training-induced enhancement of synaptic structure and function, we measured the levels of several synaptic proteins by Western blotting. The results showed that spatial training induced upregulation of postsynaptic proteins glutamate AMPA receptor GluA1 subunit (GluA1), PSD93 and PSD95 with not obvious effects on the levels of vesicle associated membrane protein 2 (Vamp2), synaptophysin (Syn), N-Methyl-D-aspartate receptor 2A and 2B subunit (GluN2A, GluN2B) in Tg2576 mice (Fig. 5a and b).

To further explore the mechanisms underlying the training-induced upregulation of postsynaptic proteins, we measured the level and the activity-dependent phosphorylation of CaMKII, cAMP-dependent protein kinase (PKA) or extracellular signal-related kinase 1/2 (ERK1/2), which can regulate synaptic plasticity^25^,^26^,^27^. The results showed that level of the phosphorylated CaMKII at Thr286 (pCaMKII) was remarkably increased in hippocampus of Tg2576 mice after training, whereas the levels of total CaMKII, total ERK1/2 and pERK1/2, PKAα catalytic subunit (PKAα-cat), and PKA Iβ regulatory subunit (PKA Iβ-reg) were not changed (Fig. 5c and d). These data suggest that CaMKII activation may play a role in spatial training-induced dendrite remodeling.

### Spatial training decreases the levels of Aβ and tau phosphorylation

**Figure 6:**
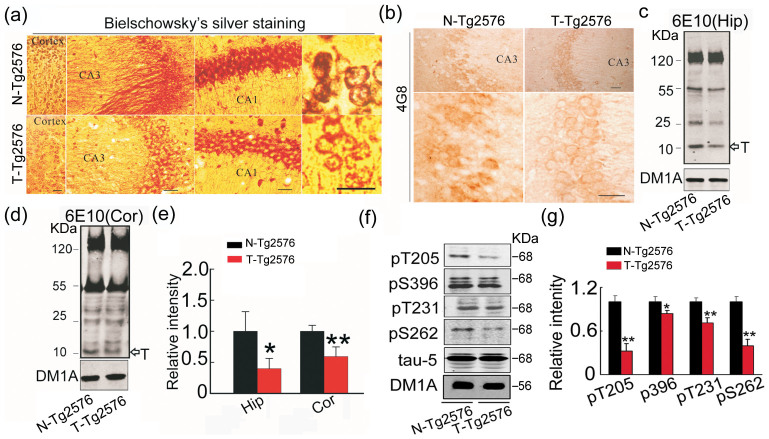
Spatial training decreases Aβ level and attenuates tau phosphorylation. (a) The representative silver staining in hippocampus and the cortex of ~9 m old Tg2576 mice with and without spatial training. (b) Aβ level in hippocampal CA3 probed by 4G8 antibody. (c–e) Aβ levels in hippocampus (Hip) (c) and cortex (Cor) (d) of Tg2576 mice measured by Western blotting and quantitative analysis (e). Data were expressed as mean ± SD (n = 4 each group). Bar = 50 μm. (f, g) The representative Western blots and quantitative analyses show tau phosphorylation levels at multiple AD-related sites in ~9 m old Tg2576 mice with or without spatial training. Blot images were cropped for comparison. DM1A was used as a loading control in each sample. The optical density of bands was quantified by Gel-Pro Analyzer 4 (Toyobo, Osaka, Japan) software. Data were expressed as mean ± SD (n = 4 each group). Student's t test was used. (e) p_Hip_ = 0.032, p_Cor_ = 0.006. (g) p_205_ < 0.001, p_396_ = 0.036, p_231_ = 0.031, p_262_ < 0.001.

We also observed that spatial training decreased Aβ level (Fig. 6a–e) with reduced tau phosphorylation at several AD-related sites in the hippocampus of Tg2576 mice (Fig. 6f and g). These changes in pathogenic Aβ and/or tau levels could also contribute to the improved memory by spatial training.

## Discussion

In the present study, we found in Tg2576 mice that spatial training could improve recent and remote memory with enhancement of dendritic complexity and spine generation. Previous studies have shown that environmental enrichment or learning can delay the development of neuropathology or memory decline in 3 × Tg-AD mice^8^,^28^, our current findings give new insights for the synaptic mechanisms underlying the spatial training-induced improvement of the cognitive functions.

Tg2576, htau and 3 × Tg-AD transgenic mice have been widely used as AD models. The htau (human full length tau) transgenic mice show visuospatial learning impairment at 6 m^29^, abnormal neuronal morphology at 13 m and cognitive deficits in MWM at 12 m old^30^,^31^. The accumulation of the hyperphosphorylated tau in hippocampus was detected at 3 m, and paired helical filaments were observed at 9 m in htau mice. The 3 × Tg-AD mice (homozygous for the PS1 mutation, APPSwe and tauP301L transgenes) show learning and memory deficits in MWM or contextual fear conditioning, formation of the extracellular Aβ plaques in the cerebral cortex and tau pathology at 6 m old^32^, and tangle pathology was advanced by 20 m^33^. The Tg2576 mice show dendrite impairments at 6 m, mild cognitive impairment at 8 m, and Aβ and tau pathologies with severe learning and memory deficits at 12 m^34^,^35^. These mice models have been widely employed for studying tau or/and Aβ pathologies. In the current study, we used 8 month old Tg2576 mice to study whether spatial training could attenuate Aβ-induced neuronal dendrite and spine impairments and thus improve the cognitive ability of the mice. We found that spatial training significantly improved the memory acquisition (48 h after the last MWM training) and the maintenance (28 days after the last MWM training).

A previous study in 3 × Tg-AD mice showed that learning can significantly reduce the plaque loads in the brain^8^. In the current study, we also observed that spatial training could reduce amyloid and tau pathologies. Although both abnormal tau and amyloid are implicated in memory deficits of AD, the direct link between these pathologies and the machineries related to cognition is still lacking. In AD patients, loss of neuronal processes is a major cause of cognitive impairment. The shape of dendritic arbor determines the total synaptic input a neuron can receive^36^, and influences the types and distribution of these inputs^37^. In the present study, we observed severe impairments of apical and basal dendritic arbors in hippocampus of ~9 m old Tg2576 mice, and spatial training significantly enhanced the dendrite complexity with enhanced LTP. These morphological and functional enhancements can facilitate learning and memory.

The postsynaptic spine remodeling is fundamental for memory formation. The recent and remote memory is associated with time-dependent formation of dendritic spines in the hippocampus and anterior cingulated cortex^38^. One of the most remarkable features of dendritic spines is their morphological diversity that endows the spines with different functional properties^39^. The large mushroom-like spines contain stacks of smooth endoplasmic reticulum and dense-staining plates that are important in regulating local calcium^40^. The newly synthesized GluA1 can be selectively recruited to mushroom-type spines in adult hippocampal CA1 neurons 24 h after fear conditioning, which demonstrates a critical functional distinction in the mushroom spines with learning^41^. On the other hand, the thin spines maintain the structural flexibility that can accommodate new, recently enhanced or weakened inputs, making them candidates for being ‘training spines’ or ‘learning spines’, whereas the stability of mushroom spines suggests that they are ‘memory spines’. Long-term potentiation induces ‘learning spines’ into ‘memory spines’, and long-term depression converts ‘memory spines’ into ‘learning spines’^41^. Our data show that the total spine density is decreased significantly in Tg2576 that is in accordance with previous report^42^. More importantly, we find that both mushroom and thin spines are increased by spatial training, but the increase of thin morphology is more prominent, suggesting that the training increases the plasticity of the dendritic spines.

The dendrite morphology is regulated by neuronal activity^43^, and many neuro-regulating molecules, such as semaphorin 3A^44^, neurotrophins^45^, Slit-1^46^, and cadherins and their signaling partner β-catenins^47^. Many protein components of the post-synaptic density (PSD) are also key regulators of the structure and function of dendritic spines in the brain^16^. We found that spatial training increased levels of GluA1, PSD93 and PSD95 with no obviously influence on synaptophysin, Vamp2, GluN2A and GluN2B. Therefore, we measured the expression levels and the activity-dependent phosphorylation of CaMKII, PKA and ERK1/2, which are involved in dendrite remodeling^25^,^26^,^27^. Interestingly, spatial training only increased the level of pCaMKII (active form) with not effects on PKA and ERK1/2 in the mice. Previous studies show that synaptic activity can activate CaMKII and regulate its postsynaptic localization^48^, which in turn activate CaMKII at postsynaptic sites and facilitate synaptic transmission by phosphorylating and promoting synaptic insertion of α-amino-3-hydroxy-5-methyl-4-isoxazole-propionic acid (AMPA) receptors^49^. CaMKII can also regulate the formation, growth and branching of dendrites^17^. The activation of CaMKII may explain the increased postsynaptic proteins observed in our current study.

The contextual fear conditioning contains a major anxiety component, while the MWM task may induce important levels of anxiety. Therefore the effect observed in the contextual fear conditioning task might be due to a better strategy obtained by MWM training to deal with stress but not due to the cognitive improvement. To minimize this possibility, we used same swimming time in three groups of mice. In future studies, less stressful spatial training paradigms, such as Barnes maze training, could be applied.

### Conclusion

We conclude that spatial training can improve both recent and remote memory in Tg2576 mice, and remodeling of dendrite complexity and spine generation may underlie the cognitive training-induced memory improvement.

## Methods

### Mice

Tg2576 mice harboring the human amyloid precursor protein 695 with Swedish double mutation (hAPP) (HuAPP695; K670N/M671L) were purchased from Jackson Laboratory and bred in the Experimental Animal Central of Tongji Medical College, Huazhong University of Science and Technology. After weaning, the mice were housed (4 to 6 mice per cage) with free access to food and water under a 12:12 hr reversed light-dark cycle, with light on at 8:00 pm. A total of 72 tg2576 mice (46 male, 26 female) and 40 wild type C57 mice were included at the beginning of the study. 2 mice died over the course of the study. All animal experiments were performed according to the “Policies on the Use of Animals and Humans in Neuroscience Research” revised and approved by the Society for Neuroscience in 1995, and the animal study was approved by the Academic Review Board of Tongji Medical College.

### Spatial training paradigm

The standard Morris water maze (MWM) procedure with minor modifications was used for the spatial training^50^. Briefly, the mice were trained in MWM to find the hidden platform for six consecutive days and four trials per day with a 30 min interval. During the training, each mouse was placed into the water by hand, so that it faced the wall of the pool, at one of four starting positions. The animals were not allowed to search the platform for more than 60 s, after which they were guided to the platform and placed on the platform for 30 s. In each trial, the swimming path and the latency to locate the hidden platform were recorded using Noldus video tracking system (Ethovision). The control groups were handled with swimming activity but without training (have the same swimming time as the training mice in the maze without the platform). At 24 h after spatial training in MWM, one part of mice were sacrificed to execute the biochemistry experiment, electrophysiological analysis and Golgi staining, the others were used to detect memory abilities by contextual fear conditioning. All experiments were conducted and analyzed by the experimenters blind to the grouping of the animals.

### Contextual fear conditioning

To test the effect of spatial training on the hippocampus-dependent associative memory, contextual fear conditioning^51^ was used.

During the training phase of the fear conditioning, the animals were placed in the conditioning chamber for 3 min, then were subjected to 3 min unsignalled foot-shocks (one shock at the first min, three shocks at the second min and 8 shocks at the third min; 0.5 mA, 2-sec duration, and 1 min apart). After the last shock, mice were remained in the chamber for 1 min, and then returned to their home cages. During the testing phase, the mice were placed back into the conditioning chamber for 3 min. At the end of each session, mice were returned to their home cages and the chambers were cleaned with water after 70% ethanol wiping. The conditioning chamber had a plexiglass front and gray side- and back-walls (width × depth × height; 175 × 165 × 300 mm), and the chamber floors were consisted of 26 stainless steel rods with a diameter of 2 mm diameter placed 5 mm apart. The rods were connected to a shock generator via a cable harness. All experiments were conducted using a video tracking system to measure the activity and freezing behavior of the animals. Freezing was defined as a complete absence of movement, and the duration of the freezing response was scored at 1 sec after the sustained freezing behavior. For each testing session, freezing (%) was then averaged among mice within same group.

### Electrophysiological analysis

Animals were anesthetized with 6% chloral hydrate, then the brain slices were cut (300 μm) in ice cold artificial cerebrospinal fluid (aCSF) after removed from skull. The composition of aCSF was (in mM): NaCl 124; KCl 3.0; MgCl_2_ 1.0; CaCl_2_ 2.0; NaH_2_PO_4_ 1.25; NaHCO_3_ 26; glucose 10; saturated with 95% O_2_, 5% CO_2_ (pH 7.4). Individual slices were laid down over an 8 × 8 array of planar microelectrodes, each 50 × 50 μm in size, with an interpolar distance of 450 μm (MED-P5455; Alpha MED Sciences, Kadoma, Japan) and kept submerged in aCSF (4 ml/min; 30°C) with a nylon mesh glued to a platinum ring. Voltage signals were acquired using the MED64 System (Alpha MED Sciences). fEPSPs were obtained by stimulating the Schaeffer collateral-commissural fibers. Stimulation intensity was adjusted to evoke fEPSP amplitudes that were 40% of maximal size. Long term potentiation (LTP) was induced by applying one train of high-frequency stimulation (HFS; 100 Hz, 1 s duration at test strength).

### Golgi impregnation

Golgi-Cox impregnation was performed by GD Rapid Golgi Stain Kit (FD Neurotechnologies). Briefly, at 24 h after spatial training in MWM, the animals (n = 4 ~ 6 per group) were sacrificed by overdose of 6% chloral hydrate, and perfused for 5 min with PBS, followed by 4% PFA in PBS for 15 min. Brains were immersed in impregnation solution (equal volumes of Solutions A and B, containing mercuric chloride, potassium dichromate, and potassium chromate), and stored at room temperature. Impregnation solution was replaced after 24 h. After 2 weeks, brains were transferred to Solution C and stored at 4°C for 48 h, with the solution replaced after 24 h. The brain was sectioned sagittally (100 μm) using a vibrate microtome (VT 1000 s, Leica) and sections were mounted on gelatin-coated microscope slides with Solution C. Slides were rinsed twice in distilled water (2 min each), and then placed in a mixture of Solution D: E: distilled water (1:1:2) for 10 min. After rinsing with distilled water, sections were dehydrated in 50%, 75%, 95% and 100% ethanol for four times (4 min each). Sections were cleared in xylene three times (4 min each) and coverslipped with Permount solution.

### Dendrite and spine analyses

The dendrites selected for quantitative analyses must meet the following criteria: (i) the cells must have a universal and even impregnation from the cell body to the tertiary branches; (ii) the dendrite must not be at an angle with the image plane because these tilted dendrites would introduce errors in measuring the length of the dendrite; and (iii) the dendrite must be well separated from other dendrites to avoid confusion in which spines that actually belong to other dendrites are counted^24^. A digital camera system and Image software were used for morphometric analysis of digitized images. Using the center of the soma as reference point, dendritic length and branch points were measured as a function of radial distance from the soma by adding up all values in each successive concentric segment (Sholl's analysis)^21^. For each animal, at least 36 neurons were analyzed.

Total dendritic length, number of branch points, and the number of primary dendrites were analyzed for every neuron. In addition, spine density was determined in two segments of dendrites at a distance of 90–110 μm (proximal) and 190–210 μm (distal) from the soma. When counting different types of spines, we counted mushroom-shaped spines with well formed head and neck structures vs. thin spines and filopodia-like structures^23^. Spines with a head diameter of at least ≈0.3 μm were categorized as mushroom types^24^. Spine quantification was carried out by observers who were unaware of the experimental conditions.

### Western blotting

Western blotting was carried out as described previously^52^. Mice were decapitated after the spatial memory retention test. The hippocampus were rapidly removed and homogenized at 4°C using a Teflon glass homogenizer with 50 mM Tris-HCl (pH 7.4), 150 mM NaCl, 10 mM NaF, 1 mM Na_3_VO_4_, 5 mM EDTA, 2 mM benzamidine, and 1 mM phenylmethylsulfonyl fluoride. The extract was mixed with sample buffer (3:1, v/v) (200 mM Tris-HCl (pH 7.6), 8% SDS, 40% glycerol, and 40 mM dithiothreitol) boiled for 10 min, and then the mixture were stored at −80°C. The proteins were separated by 10% SDS-polyacrylamide gel electrophoresis and transferred to nitrocellulose membranes. The membranes were blocked with 5% nonfat milk dissolved in TBS Tween-20 (50 mM Tris-HCl (pH 7.6), 150 mM NaCl, 0.2% Tween-20) for 1 h and probed with primary antibody at 4°C overnight. Finally, the blots were incubated with anti-rabbit or anti-mouse IgG conjugated to IRDye™ (800CW) for 1 h and visualized using the Odyssey Infrared Imaging System (Licor biosciences, Lincoln, NE, USA). The antibodies used in the current study are listed in Table 1. Densitometry analysis was performed by measuring the optical densities of the targeted protein bands relative to the DM1A level from the same brain sample. The analysis was performed using Gel-Pro Analyzer 4 (Toyobo, Osaka, Japan) software.

### Immunohistochemistry

Animals were anesthetized with 6% Chloral hydrate, fixed in situ, and perfused through the aorta with 100 ml 0.9% NaCl, followed by 400 ml phosphate buffer containing 4% paraformaldehyde. The brain was postfixed in perfusate for 24 h and coronal sections from the hippocampus were cut at 25 μm with a vibratome (VT1000S, Leica, Nussloch, Germany). The sections of mouse brain were collected consecutively in PBS for immunohistochemical staining. The vibratome sections were permeabilized for 30 min to block endogenous peroxidase and nonspecific sites were blocked with bovine serum albumin for 30 min at room temperature. Sections were then incubated overnight at 4°C with primary antibodies. After washing with PBS, sections were subsequently incubated with biotin-labeled secondary antibodies at 37°C for 1 h. The immunoreaction was detected using horseradish peroxidase-labeled antibodies at 37°C for 1 h and visualized with the diaminobenzidine tetrachloride system (brown color). For each primary antibody, 3 to 5 consecutive sections from each brain were used. The images were observed using a microscope (Olympus BX60, Tokyo, Japan).

### Statistical analysis

The data were expressed as mean ± SD and analyzed by the one-way analysis of variance procedure followed by least significant difference post hoc tests or student's t tests using SPSS 12.0 statistical software (SPSS Inc., Chicago, Illinois). A *p* value of <0.05 was considered as statistically significant in all experiments.

## Author Contributions

Author contributions: G.P.L. and J.Z.W. designed research; X.J., S.G.C., Z.H.W., Y.H., X.C.L., Z.W.M. and Q.W. performed research; X.J., G.P.L. and J.Z.W. analyzed data, G.P.L. and J.Z.W. wrote the paper.

## Supplementary Material

Supplementary Information Supplementary information

## Figures and Tables

**Table 1:** Antibodies employed in the study

Antibody	Specific	Type	WB	IH	Source
pT205	Phosphorylated tau at Thr205	pAb	1:1000		SAB
pT231	Phosphorylated tau at Thr231	pAb	1:1000		SAB
pS262	Phosphorylated tau at Ser262	pAb	1:1000		SAB
pS396	Phosphorylated tau at Ser396	pAb	1:1000		Biosource
tau-5	Total tau	pAb	1:1000		Millipore
Vamp2	vesicle associated membrane protein 2	pAb	1:1000		Millipore
Syp	Total synaptophysin	mAb	1:1000		Cell Signaling
PSD95	Total post synaptic protein 95	mAb	1:1000		Millipore
PSD93	Total post synaptic protein 93	mAb	1:1000		Abcam
GluA1	Total GluA1	pAb	1:1000		Millipore
GluN2A	Total GluN2A	pAb	1:1000		Millipore
GluN2B	Total GluN2B	pAb	1:1000		Millipore
PKAα	PKA catalytic subunit	pAb	1:1000		Santa Cruz
PKAβ	PKA regulatory subunit	pAb	1:1000		Santa Cruz
ERK1/2	Total p44/42 mitogen-activated protein kinase	mAb	1:1000		Cell Signaling
pERK1/2	Phosphorylated ERK	pAb	1:1000		Cell Signaling
CaMKII	Total CaMKII	pAb	1:1000		Cell Signaling
pCaMKII	phosphorylated CaMKII at Thr286	pAb	1:1000		Cell Signaling
6E10	Aβ_1–16_	mAb	1:1000		Signet
4G8	Aβ_17–24_	mAb		1:1000	Signet
DM1A	α-tubulin	mAb	1:1000		Sigma

WB: Western blotting; IH: immunohistochemistry.
