# Supplementary material for: Spatial training preserves associative memory capacity with augmentation of dendrite ramification and spine generation in Tg2576 mice
Source: Sci Rep. 2015 Mar 30;5:9488. doi: 10.1038/srep09488 (PMC4377552; doi:10.1038/srep09488)
Supplement: Supplementary Information [file srep09488-s1.doc]

Spatial training preserves associative memory capacity with augmentation of dendrite ramification and spine generation in Tg2576 mice

Xia Jiang, Gao-Shang Chai, Zhi-Hao Wang, Yu Hu, Xiao-Guang Li, Zhi-Wei Ma, Qun Wang, Jian-Zhi Wang, Gong-Ping Liu

**SUPPLEMENTARY INFORMATION**

**
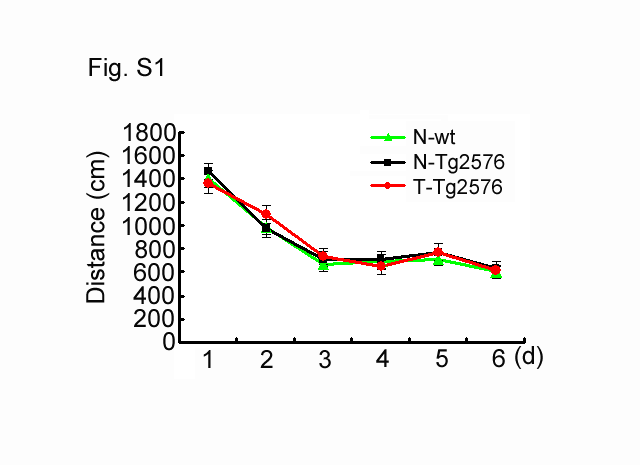
**

**Supplementary Figure 1. No significant difference in swimming distance was detected in three groups of mice.** Tg2576 mice were trained for 6 consecutive days training (T-Tg2576) in the Morris water maze, while the control Tg2576 (N-Tg2576) and the non-Tg littermates (N-wt) received the same swimming time as the training mice in the maze, the swimming distance of each day in the maze were measured. Data were expressed as mean±SD (n = 10~12 each group). One-way ANOVA followed by post hoc tests (LSD) was used.
